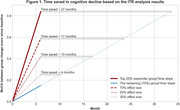# How Much Time Can be Saved in Cognitive Decline? the Internet‐based Conversational Engagement Clinical Trial (I‐CONECT)

**DOI:** 10.1002/alz.086844

**Published:** 2025-01-09

**Authors:** Chao‐Yi Wu, Kexin Yu, Steven E. Arnold, Sudeshna Das, Hiroko H Dodge

**Affiliations:** ^1^ Massachusetts General Hospital, Harvard Medical School, Boston, MA USA; ^2^ NIA‐Layton Aging & Alzheimer’s Disease Research Center, Oregon Health & Science University, Portland, OR USA

## Abstract

**Background:**

Clinical trials should strive to yield results that are clinically meaningful rather than solely relying on statistical significance. However, the determination of clinical meaningfulness of dementia clinical trials lacks standardization and varies based on the trial’s nature. To tackle this issue, a proposed approach involves assessing the time saved before reaching a specific threshold in cognitive status. In this study, we investigated the time saved in cognitive decline among the top responders based on the individual‐level treatment responses (ITR) analysis, using data from the Internet‐based Conversational Engagement Clinical Trial (I‐CONECT; NCT02871921).

**Method:**

I‐CONECT is a randomized controlled trial to examine the effects of conversational interactions on cognition among socially isolated participants aged ≥ 75 years old. The experiment group engaged in video chats with study staff 4 times/week for 6 months; the control groups received weekly check‐in phone calls. We focused on cognitive outcomes that exhibited significant treatment effects at 6‐month follow‐up: the Montreal Cognitive Assessment (MoCA) for global cognition and Category Fluency Animals (CFA) for semantic fluency. To assess ITR, we employed 300 iterations of 3‐fold cross‐validated random forest models. We estimated treatment heterogeneity by conducting permutation tests on the area between curves (ABC) statistics derived from the ITR scores. We estimated time saved in cognitive decline as the difference in the number of months required for the top responders (top 25%; 33%) and the remaining participants to reach the same cognitive level at 6‐months follow‐up.

**Result:**

ABC statistics showed substantial heterogeneity in treatment response with MoCA but modest heterogeneity in treatment response with CFA. For global cognition, assuming a treatment effect size between 30‐50%, the top 25% and 33% of responders exhibited potential cognitive delays ranging from 4.1 to 10.8 months and 5.9 to 13.9 months, respectively (Figure 1). For semantic fluency, large effect sizes (70‐100%) are required to show potential cognitive delays ranging from 0.5 to 6.7 months.

**Conclusion:**

Individual differences in the time saved for cognitive decline through the ITR analysis are meaningful outcomes in clinical trials. Future trial outcomes should consider both quantity and quality concepts such as quality‐adjusted time saved.